# Epidemiology and long-term prognosis of atrial fibrillation in rural African patients

**DOI:** 10.1186/s43044-019-0005-3

**Published:** 2019-09-05

**Authors:** Dakaboué Germain Mandi, Joel Bamouni, Dangwé Temoua Naïbé, Rélwendé Aristide Yaméogo, Elisé Kaboré, Yibar Kambiré, Koudougou Jonas Kologo, Georges Rosario Christian Millogo, Nobila Valentin Yaméogo, Anna Thiam Tall, Patrice Zabsonré

**Affiliations:** 1 Cardiology Unit, Department of General Medicine, Regional Hospital Center of Tenkodogo, Tenkodogo, Burkina Faso; 2 Superior School of Health Sciences, University of Ouahigouya, Ouahigouya, Burkina Faso; 3 Faculty of Human Health Sciences, University of N’Djamena, N’Djamena, Chad; 4 University of Normandie, UNIHAVRE- UNIROUEN - UNICAEN, CNRS, UMR IDEES, Le Havre, France; 5 Training and Research Unit of Health Sciences, University Ouaga I - Professeur Joseph Ki-Zerbo, Ouagadougou, Burkina Faso; 6 Department of cardiology, Teaching Hospital of Yalgado Ouedraogo, Ouagadougou, Burkina Faso

**Keywords:** Atrial fibrillation, Epidemiology, Anticoagulation, Morbidity, Mortality, Africa

## Abstract

**Background:**

Few studies have addressed the pattern of atrial fibrillation (AF) in rural Africa. The purpose of the study was to assess the epidemiology and long-term prognosis of AF in rural African patients in the Regional Hospital Center (RHC) of Tenkodogo, Burkina Faso.

**Results:**

Overall, 107 of 1805 cardiac cases presented with AF (prevalence of 5.9%). Six patients were excluded. Mean age was 66.56 ± 14.92 years, and 53.47% were female. Hypertension was the most prevalent cardiovascular risk factor (59.41%). Congestive heart failure (HF) was reported in 85.15% of the study patients at presentation. Most of the study population presented with severe underlying heart disease (93.1%), and hypertensive heart disease was the most prevalent with 45.54% of the cases. The mean CHA_2_DS_2_VASc score in patients with non-valvular heart disease (*n* = 91) was 3.33 ± 1.25 (extremes 1–6) while the risk of bleeding was low (HAS-BLED score ≤ 1) in 82 patients (81.2%). Oral anticoagulation was prescribed in few cases (5.26%). During a follow-up period of 74.43 ± 23.94 weeks, acute HF and stroke occurred in respectively 43 and 6 patients. Forty-one patients (40.59%) died. The overall survival rate was 69% at 6-month and 59.4% at 1-year follow-up. Patients with idiopathic dilated cardiomyopathy were at higher risk of death than other patients (log-rank test = 11.88, *p* < 0.001) over time.

**Conclusion:**

AF is not rare in rural African patients and is associated with an increased long-term risk of HF, stroke, and mortality.

## Background

Sub-Saharan Africa (SSA) is facing an epidemiological transition with an exponential increase of non-communicable diseases particularly cardiovascular ones such as atrial fibrillation (AF). AF is the most common sustained arrhythmia in clinical practice occurring in patients with a variety of cardiovascular and non-cardiac conditions, rising in incidence with patient age to affect nearly one quarter of the adult population over their lifetime [1–3]. Its prevalence is globally increasing [2, 3]. Hospital-based studies revealed that the prevalence of AF in SSA is ranging from 4.6 to 10.6% [4–7]. AF is strongly associated with an increased morbidity, such as stroke/systemic embolism or heart failure [8–11], and high risk of mortality [2, 11]. Healthcare systems in SSA are also characterized by limitations in human and financial resources and geographical access [12]. Data on AF in Burkina are scarce and only available for urban centers. Therefore, the present study aimed to assess the long-term prognosis of AF in rural Africans in the Eastern Center region of Burkina Faso, Western Africa.

## Patients and methods

This prospective cohort study was conducted in the cardiology unit of the department of medicine of the Regional Hospital Center (RHC) of Tenkodogo, Tenkodogo, Burkina Faso. The RHC of Tenkodogo is the tertiary healthcare center that covers a dry orchard savannah region populated of about 1.4 million inhabitants, almost constituted by subsistence farmers. Tenkodogo, the capital town of this eastern-center region, is located at 188 km from Ouagadougou, the political capital town of the country. Since the opening of the RHC, the first cardiologist was assigned in 2015. So far, there is no established national health insurance in Burkina Faso and the eastern-center region makes no exception. Therefore, care costs are most of the time supported by family network. At the time of recruitment, the cardiology unit was provided with only the basic equipment such as 12-lead electrocardiogram (ECG) and transthoracic echocardiography. Ambulatory (Holter) electrocardiogram, stress test, and coronary angiography were not available. International normalized ratio (INR) was not also performed at the site and was only available at Ouagadougou.

Patients aged over 18 years attending the cardiology unit who had a documented AF on 12-lead electrocardiogram (ECG) and consented to participate were consecutively enrolled in the study from 1 January 2015 to 30 December 2016. Patients’ sociodemographic data (age, gender, socioeconomic level) were recorded. Lower socioeconomic status was defined as an average income < 2 US dollar per day [13]. Cardiovascular risk factors (hypertension, mellitus diabetes, dyslipidemia, smoking, obesity, alcohol use), anthropometric parameters, signs, and symptoms were collected. Patient’s serum creatinine, glucose, uric acid, ions, and hemoglobin were measured and thyroid hormones checked when clinically indicated. Two-dimensional and Doppler transthoracic echocardiography was used for the diagnosis of underlying heart disease. Left ventricular ejection fraction was measured using Simpson biplane formula [14]. We did not classify AF into its different types (new onset, paroxysmal, persistent, and permanent) due to lack of previously recorded ECGs at the time of presentation. Lone AF refers to episodes of AF in patients younger than 65 years without clinical and echocardiographic evidence of underlying cardiac disease.

**Fig. 1:**
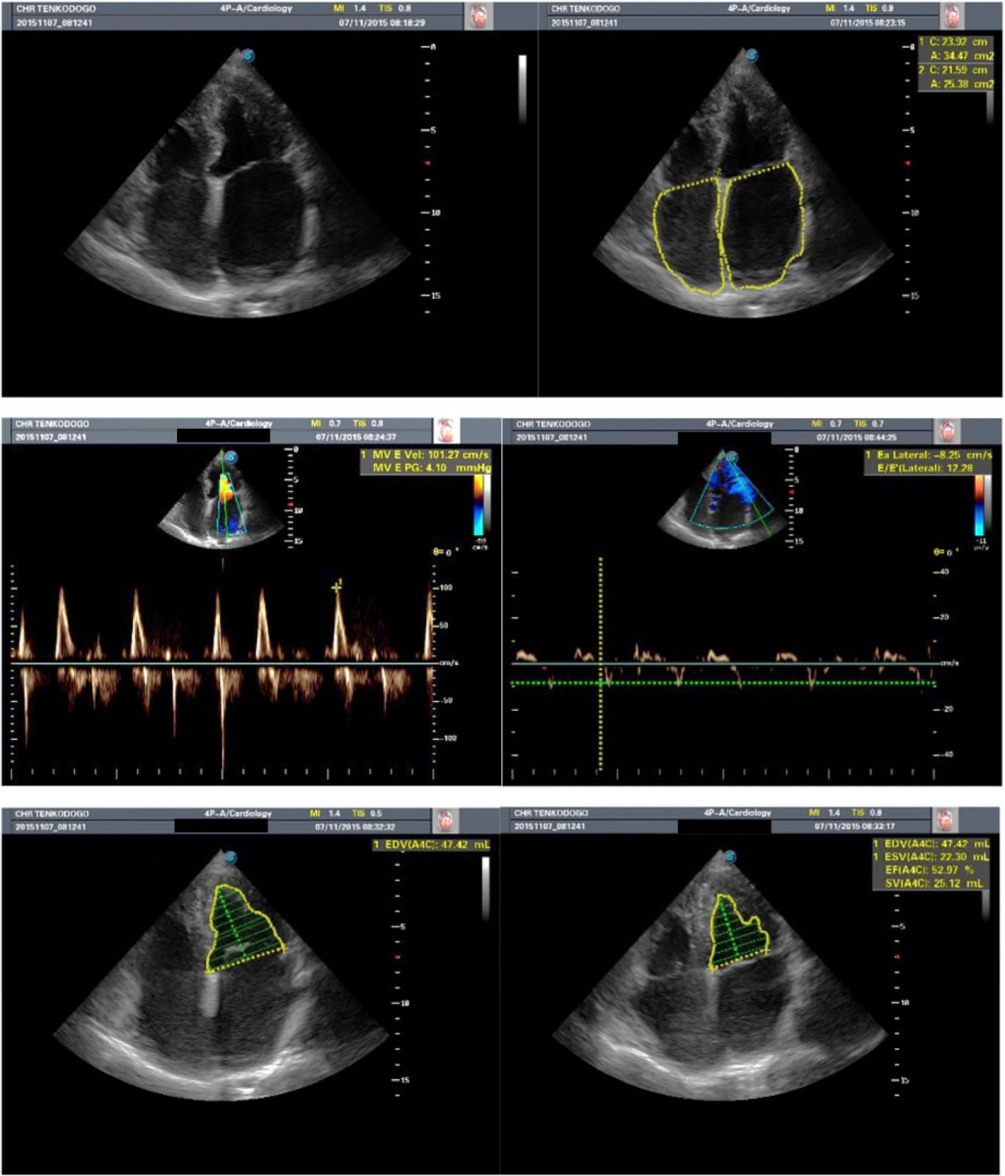
Echocardiogram revealing restrictive cardiomyopathy in a 78-year-old woman with atrial fibrillation. Note the reduction of both ventricles’ volumes, normal left ventricle (LV) wall thickness and massive bi-atrial enlargement in apical four-chamber views. The LV ejection fraction was slightly reduced with evidence of diastolic dysfunction

In practice, the diagnosis of hypertensive heart disease was based on the presence of echocardiogram abnormalities such as left ventricular hypertrophy (concentric or eccentric), increased left ventricular mass index, enlarged left ventricular or left atrial size, and increased volumes, and diastolic or systolic left ventricular dysfunction in patients with hypertension apart from obvious alternative explanation. Ischemic heart disease was diagnosed in patients with either history of typical angina or acute myocardial infarction, and/or typical ECG abnormalities of acute myocardial infarction or myocardial ischemia, plus ventricular regional wall motion abnormality on 2D echocardiography. Valvular heart disease diagnosis was based on joint ESC/EACTS guidelines on the management of valvular heart disease [15]. Restrictive cardiomyopathy was considered in the presence of normal or reduced diastolic volumes (of one or both ventricles), normal or reduced systolic volumes, and normal ventricular wall thickness [16] as shown in Fig. 1. Idiopathic dilated cardiomyopathy was considered in the presence of left ventricular dilatation and left ventricular systolic dysfunction in the absence of abnormal loading conditions (hypertension, valve disease), coronary artery disease, congenital heart disease, or any obvious clinical condition sufficient to cause global systolic impairment. Right ventricular dilation and dysfunction may be present but are not necessary for the diagnosis [16].

The CHA_2_DS_2_VASc score was used to stratify the thrombo-embolic risk in patients with non-valvular heart disease (VHD), both rheumatic and degenerative. The risk was low for a score of 0, intermediate for a score of 1–2, and high for a score > 2 [17]. The hemorrhagic risk was assessed in all patients using HAS-BLED score. This risk was low for a score ≤ 1, intermediate for a score of 2–3, and high for a score ≥ 4 [18]. Prescribed medications were also assessed.

Patients were followed up until 30 June 2017. Outcomes of interest were ischemic stroke, heart failure (HF), and death. The immediate cause of death was also recorded.

Data were analyzed using Epi info 7 version 3 and R software. Continuous variables were expressed as means ± standard deviation (SD) and categorical variables as percentage. Differences between groups were assessed using chi-square or Student test as appropriate. Survival curves were performed using the Kaplan-Meier method and log-rank test used for comparison. A value of *p* < 0.05 was considered statistically significant.

## Results

During the recruitment period, 1805 patients attended the cardiology unit (both outpatients and inpatients). AF was found in 107 patients accounting for 5.9% of all admissions. Six patients were excluded from the final analysis process (uncomplete data 04, lost to follow-up 02). Of the remaining 101 patients, 47 were male (46.53%) and 54 were female (53.47%). Their mean age was 66.56 ± 14.92 years (extremes 20–99 years). Most of the study patients were of low socioeconomic condition (89.11%). Hypertension was the most prevalent cardiovascular risk factor (59.41%). Congestive HF was reported in 85.15% of the study patients at presentation (Table 1).

**Table 1:** Clinical baseline characteristics of the study population

Characteristic	Value
Age (years), mean ± SD	66.56 ± 14.92
Female, *n* (%)	54 (53.47)
Low socioeconomic level, *n* (%)	90 (89.11)
Cardiovascular risk factors	
Hypertension, *n* (%)	60 (59.41)
Diabetes, *n* (%)	07 (6.90)
Smoking, *n* (%)	29 (28.71)
Dyslipidemia, *n* (%)	04 (3.96)
Alcohol abuse, *n* (%)	12 (11.88)
Body mass index (kg/m^2^), mean ± SD	21.63 ± 3.17
Obesity (body mass index ≥ 30 kg/m^2^), *n* (%)	3 (2.97)
Heart rate (beats/minute), mean ± SD	108.99 ± 29.08
Systolic blood pressure (mmHg), mean ± SD	126.12 ± 28.89
Diastolic blood pressure (mmHg), mean ± SD	76.73 ± 16.25
Signs and symptoms	
Congestive heart failure, *n* (%)	86 (85.15)
Palpitations, *n* (%)	59 (58.42)
Chest pain, *n* (%)	30 (29.70)

Underlying heart disease was found in 94 patients (93.1%). Hypertensive heart disease was present in 45.54% of the cases. Mean left ventricular ejection fraction (LVEF) was 41% ± 17. HF with reduced ejection fraction (LVEF < 40%) was reported in 60 patients (Table 2).

**Table 2:** Laboratory baseline findings and underlying heart diseases of the study population

Parameter	Value
Serum glucose (mmol/l), mean ± SD	4.68 ± 1.31
Hemoglobin (g/dl), mean ± SD	11.74 ± 1.97
Serum creatinine (μmol/l), mean ± SD	123.1 ± 56.48
eGFR (ml/min/1.73 m^2^), mean ± SD	61.67 ± 24.66
Uric acid (μmol/l), mean ± SD	387.81 ± 101.54
Serum potassium (mmol/l), mean ± SD	3.97 ± 0.49
Serum calcium (mmol/l), mean ± SD	2.1 ± 0.23
Sodium (mmol/l), mean ± SD	135.55 ± 8.28
QRS duration (milliseconds), mean ± SD	110.35 ± 9.28
Left bundle block branch, *n* (%)	13 (12.87)
Left atrium diameter (mm), mean ± SD	46.04 ± 7.06
Left atrium surface (cm^2^), mean ± SD	23.73 ± 6.50
LVEF (%), mean ± SD	41.4 ± 17
LVEF < 40%, *n* (%)	60 (59.41)
LVEF = 40–49%, *n* (%)	9 (8.91)
LVEF ≥ 50%, *n* (%)	32 (31.68)
Left ventricular filling pressure (E/E’), mean ± SD	14.68 ± 4.65
Left ventricular diastolic dysfunction, *n* (%)	75 (74.26)
Heart diseases and etiologies	
Hypertensive heart disease, *n* (%)	46 (45.54)
Idiopathic dilated cardiomyopathy, *n* (%)	21 (20.79)
Valvular heart disease, *n* (%)	10 (9.90)
Restrictive cardiomyopathy, *n* (%)	8 (7.92)
Ischemic heart disease, *n* (%)	6 (5.94)
Hyperthyroidism, *n* (%)	3 (2.97)
Lone AF, *n* (%)	7 (6.90)

*SD* standard deviation, *eGFr* estimated glomerular filtration rate using CKD-EPI formula, *LVEF* left ventricular ejection fraction, *AF* atrial fibrillation

Patients with non-VHD accounted for 90.1% (*n* = 91) of all study patients. Their mean age was 68.73 years versus 46.90 years in patients with VHD (*p* < 0.001). Valvular heart diseases comprised rheumatic (*n* = 6) and degenerative (*n* = 4) ones. The mean CHA_2_DS_2_VASc score on non-VHD patients was 3.33 ± 1.25 (extremes 1–6). Their risk of thrombo-embolism was intermediate (CHA_2_DS_2_VASc score of 1–2) in 25 of 91 patients (27.5%) and high (CHA_2_DS_2_VASc score > 2) in 66 of this subgroup (72.5%).

The mean HAS-BLED score was 0.95 ± 0.68 (extremes 0–3) for the all study population. The risk of bleeding was low (HAS-BLED score ≤ 1) in 82 patients (81.2%) and intermediate in 19 patients with a HAS-BLED score of 2–3.

Antiarrhythmic drugs were prescribed in 73 of 101 patients (72.28%). Amiodarone was used in 31 patients (30.69%) and digoxin in 40 patients (39.60%). None of the study patients underwent an electrical cardioversion. Anticoagulation was initiated with enoxaparin in 25 patients. Vitamin K antagonist (VKA) was prescribed in 4 of the 76 patients (5.26%) at high risk of stroke and/or systemic thrombo-embolism (66 patients with non-valvular AF and 10 patients with VHD). Antiplatelet agents were prescribed in 84 of 101 patients (83.17%). Angiotensin-converting enzyme inhibitors/angiotensin receptor blockers were used in 94 patients for the treatment of underlying conditions (Table 3).

**Table 3:** Distribution of study patients according to drug treatment

Drug	Value
Antiarrhythmics	
Amiodarone, *n* (%)	31 (30.69)
Betablockers, *n* (%)	17 (16.83)
Digoxin, *n* (%)	40 (39.60)
Calcium channel blockers, *n* (%)	02 (1.98)
Amiodarone + digoxin, *n* (%)	11 (10.89)
Antithrombotics	
Enoxaparin, *n* (%)	25 (24.75)
Vitamin K antagonist*, *n* (%)	4 (5.26)
Antiplatelet agents, *n* (%)	84 (83.17)
ACEI/ARB, *n* (%)	94 (93.07)
Spironolactone, *n* (%)	63 (62.38)
Thiazides, *n* (%)	9 (8.91)
Loop diuretics, *n* (%)	86 (85.15)
Nitrates, *n* (%)	55 (54.46)
Statins, *n* (%)	6 (5.94)

Loop diuretics and nitrates were used during the acute phase of congestive heart failure

*ACEI/ARB* angiotensin-converting enzyme inhibitors/angiotensin receptor blockers

*Four over 76 patients at high risk of stroke received VKA

**Fig. 2:**
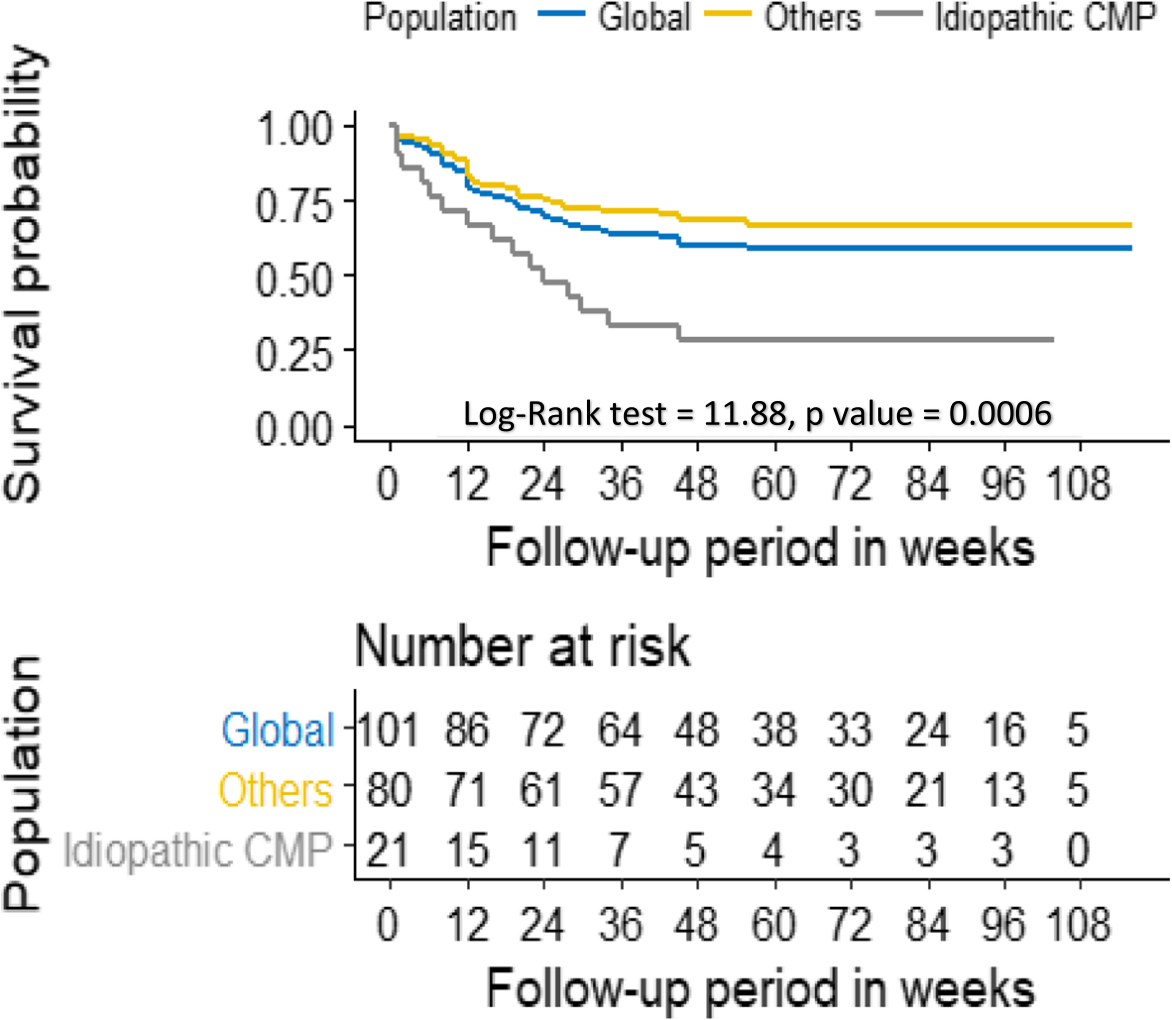
Kaplan-Meier survival curves for all population and patients stratified by the presence and absence of idiopathic cardiomyopathy (CMP)

The mean follow-up period was 74.43 ± 23.94 weeks (extremes 42–116 weeks). At the time of the last follow-up visit, 8 patients (7.92%) were in sinus rhythm. During the follow-up period, 45 patients (44.6%) were readmitted (43 patients for acute decompensation of heart failure associated with ischemic stroke in 4 cases and 2 cases of isolated ischemic stroke). No hemorrhagic complication was reported. Forty-one patients (40.59%) died. The overall survival rate was 69% at 6-month and 59.4% at 1-year follow-up. Patients with idiopathic dilated cardiomyopathy were at higher risk of death than other patients (log-rank test = 11.88, *p* < 0.001) over time (Fig. 2). The mean age of deceased patients was 70.90 ± 15.36 years compared to those who survived with a mean age of 63.60 ± 13.98 years (*p* = 0.015). Twenty-nine of the 45 readmissions (64.44%) versus 12 of the readmission-free group (21.43%) died (*p* < 0.001). The immediate cause of death was ischemic stroke (*n* = 4), acute decompensation of heart failure (*n* = 28), and sudden death defined as death that occurred unexpectedly in an otherwise stable patient within 1 h of the onset of symptoms (*n* = 9).

## Discussion

This present prospective cohort study was the first one to provide a hospital-based prevalence of AF (5.9%) in rural Burkina Faso. Previous studies in SSA have reported comparable findings however in urban populations [5, 6, 19]. In a rural Ghana, AF was reported to be scarce in a traditional African community accounting for 0.3% of 924 individuals aged of 50 years and above [20].

In developing countries, patients with AF seem to be a decade younger and more likely to have heart failure [21]. The mean age of our patients was 66.56 ± 14.92 years and is similar to that of Ntep-Gweth et al. [22] in Cameroon with 65.8 ± 13 years. Conversely, most studies in SSA reported more younger age below 60 years [4–6, 23]. It is well known that the risk of AF increases with age and is higher in patients with cardiovascular diseases and cardiovascular risk factors such as hypertension, diabetes mellitus, obesity, smoking, and alcohol abuse [24]. The majority of our AF patients had severe structural heart disease (93.1%) with hypertensive heart disease being the most prevalent and that is consistent with findings from SSA [5, 6, 22]. Hypertension was the most common risk factor of AF (59.41%) as stated by previous reports [4, 22, 25–27].

As SSA population lifespan is growing substantially, cardiovascular risk factors, particularly hypertension, are expected to significantly increase in the forthcoming decades. Consequently, there is a great need to set up strategies of prevention, treatment, and control of hypertension. Congestive HF is often the mode of presentation of AF and was highly prevalent in our cohort with 85.15%. In a prospective cohort study of 172 patients with AF conducted in Cameroon [22], congestive HF was reported in 58% of the cases. Idiopathic dilated cardiomyopathy was found to be the second most frequent underlying heart disease (20.8%) in our work. However, cardiac magnetic resonance and coronary angiography could definitely discriminate between ischemic and non-ischemic origins of this subset of dilated cardiomyopathy. Moreover, specific molecular and genetic testing should be part of the etiological diagnostic workup, as they may offer specific therapeutic opportunities [28], but they are costly for low-income countries. VHD was less frequent in our study (9.9%) compared with previous findings in SSA [4, 22]. Our low frequency of VHD could be explained by the poor outcome of such disease which is more prevalent in younger people. Consequently, their life expectancy could have been shortened especially in a context where cardiac surgery is so far not available.

In the present report, most patients had a high risk of stroke and systemic thrombo-embolism (66 patients with non-valvular AF and 10 patients with VHD) and should be prescribed oral anticoagulation (OAC) for the prevention of stoke and/or systemic embolism. Furthermore, their bleeding risk was low in the majority of the cases allowing safer anticoagulation. Only 5.26% of patients who should benefit from OAC received VKA. The OAC prescription rate in patients with AF is very contrasting but globally low in SSA [4, 7, 11, 22, 23, 29] compared with data from Europe with over 80% of eligible patients being anticoagulated [30]. Constraints on OAC prescription in our site comprised financial aspects, difficulties in monitoring INRs, particularly geographic access. Consequently, only few patients who were able to regularly travel to Ouagadougou (round trip by bus costs ≈ 8 US Dollar) could afford this biological test as most of our study patients were known to live in poor conditions. Hence, the OAC prescription rate may have been impeded leading to low physician adherence to the guidelines. Therefore, monitoring VKA treatment (INRs) should be made available and cost-effective for remote areas in SSA. It has been shown that some patients refused the initiation of VKA treatment because of repeated blood sampling for INR checks and related (nutritional diet and possible bleeding) aspects [7]. Moreover, direct oral anticoagulants are effective, have no mandatory monitoring of INRs, and could be an alternative to VKA but these are still costly for African patients. Aspirin which was widely used in our cohort (83.17%) is neither effective nor safe and has very limited indications [31].

In the present cohort study, the long-term mortality was very high (40.59%) and much higher than that observed by Ntep-Gweth et al. [22] with 29.5%. The RELY-AF Registry reported the 1-year mortality and stroke in patients from 47 countries. Over 15,000 patients were enrolled and 1750 (11%) died within 1 year. The mortality rate was significantly higher by 1 year in South America (17%) and Africa (20%) compared with North America, Western Europe, and Australia (10%, *p* < 0.001) [32]. By the end of the follow-up, ischemic stroke occurred in 6 of our patients (5.94%). Data from Africa have shown rates of 8 to 12.5% at 1 year compared with 3% in the developed world [22, 32]. Heart failure is generally the most common cause of death [32] and was reported as adverse event in 43 patients (42.6%) in the present work. Higher rates of adverse outcomes in our cohort are likely due to the low rate of OAC and the fact that these patients tend to present for cardiology care at certainly advanced stages of the underlying heart disease and thereby increasing the probability of severe complications such as heart failure and stroke and life-threatening ventricular arrhythmias.

Our study had some limitations. Undoubtedly, only patients from a privileged background who could afford cardiologist care and/or those with an advanced stage of heart disease were included. We only recruited patients attending the cardiology unit with severe cardiac diseases and therefore those with less symptomatic cardiovascular diseases or asymptomatic AF in non-cardiac units and in the community may have been excluded. Furthermore, ambulatory (Holter) ECG recording was not performed in this study for the diagnosis of silent and/or paroxysmal AF. Comparison of patients with AF to the whole population (*n* = 1805) for prognosis was not performed due to lack of data. Despite the underestimation of the burden of the disease, this work provided somewhat the spectrum of AF and highlighted the anticoagulation challenge in rural Africa.

## Conclusion

Atrial fibrillation is not rare in rural African patients and is associated with an increased long-term risk of heart failure, stroke, and mortality. Its management is challenging in rural SSA. We urged that INR check should be made available as cardiology expertise is now spreading through remote areas of Africa. It is also of great need to early detect and treat modifiable risk factors to reduce the burden of AF. Further studies are needed, particularly population-based ones, in order to characterize in deep, the spectrum of AF in this area.

## Data Availability

The dataset supporting the results and conclusions of this article will be available from the corresponding author on request.

## Abbreviations

AF: Atrial fibrillation
ECG: Electrocardiogram
eGFR: Estimated glomerular filtration rate
HF: Heart failure
INR: International normalized ratio
LVEF: Left ventricular ejection fraction
OAC: Oral anticoagulation
RHC: Regional Hospital Center
SD: Standard deviation
SSA: Sub-Saharan Africa
VHD: Valvular heart disease
VKA: Vitamin K antagonist
